# P-1765. Are we there yet? The ongoing journey for improving management of asymptomatic bacteriuria

**DOI:** 10.1093/ofid/ofae631.1928

**Published:** 2025-01-29

**Authors:** Rachel M Kenney, Satheesh Gunaga, Megan M Cahill, Lori Leman, Amy Beaulac-Harris, Erin Eriksson, Abigail Geyer, Anita Shallal, Tricia L Stein, Nicole Mazzetti, Kiera Kaiser, Janeen Dubay, Ashli Arthur, Manal Higginbottom, Namita Jayaprakash, Sairia Dass, Ayyoub Haddad, Asgar Boxwalla, Scott Kaatz, Robert Tibbetts, Michael Veve, Geehan Suleyman

**Affiliations:** Henry Ford Hospital, Detroit, Michigan; Henry Ford Wyandotte Hospital / Envision Healthcare, Wyandotte, Michigan; Henry Ford Macomb Hospital, Henry Ford Health, Clinton Township, Michigan; Henry Ford Macomb Hospital, Henry Ford Health, Clinton Township, Michigan; Henry Ford West Bloomfield Hospital, Henry Ford Health, West Bloomfield, Michigan; Henry Ford Health, Detroit, Michigan; Henry Ford Wyandotte Hospital, Henry Ford Health, Wyandotte, Michigan; Henry Ford Health, Detroit, Michigan; Henry Ford West Bloomfield Hospital, Henry Ford Health, West Bloomfield, Michigan; Henry Ford West Bloomfield Hospital, Henry Ford Health, West Bloomfield, Michigan; Henry Ford Hospital, Henry Ford Health, Detroit, Michigan; Henry Ford West Bloomfield Hospital, Henry Ford Health, West Bloomfield, Michigan; Henry Ford Jackson Hospital, Henry Ford Health, Jackson, Michigan; Henry Ford Wyandotte Hospital, Henry Ford Health, Wyandotte, Michigan; Henry Ford Hospital, Henry Ford Health, Detroit, Michigan; Henry Ford Jackson Hospital, Henry Ford Health, Jackson, Michigan; Henry Ford Wyandotte Hospital, Henry Ford Health, Wyandotte, Michigan; Henry Ford Wyandotte Hospital, Henry Ford Health, Wyandotte, Michigan; Henry Ford Hospital, Henry Ford Health, Detroit, Michigan; Henry Ford Health, Detroit, Michigan; Henry Ford Health, Detroit, Michigan; Henry Ford Health, Detroit, Michigan

## Abstract

**Background:**

Treating asymptomatic bacteriuria (ASB) with antibiotics contributes to preventable patient harm. This quality improvement study aimed to evaluate the effectiveness of ongoing diagnostic and antibiotic stewardship interventions in reducing unnecessary testing and treatment of ASB.

**Figure:**
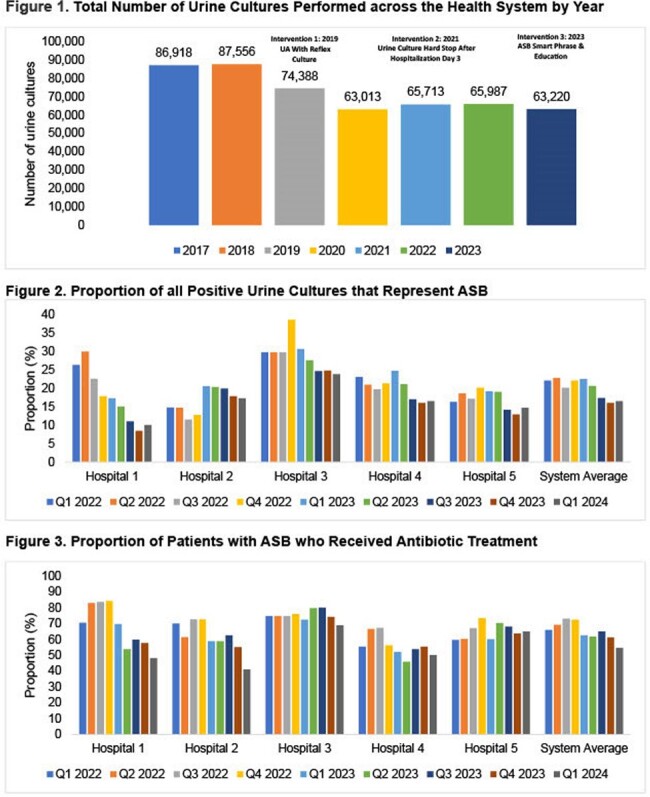

**Methods:**

This retrospective, cross-sectional quality improvement study spanned from 1/2017-3/2024 at a five-hospital urban and suburban health system in Michigan. Interventions included i). Implementation of urinalysis with reflex to urine culture in 2/2019, with reflex criteria revised in 7/2022 to ≥ 10 WBCs; ii). Adoption of a urine culture hard stop after hospitalization day 3 (3/2021); iii). Introduction of an ASB progress note smart phrase and educational initiative via a 1-page handout and narrated slides in the first quarter of 2023. The primary endpoint was the proportion of patients with ASB who received antibiotic treatment, as defined by Vaughn et al JAMA Intern Med 2023. Secondary endpoints encompassed the number of provider smart phrase documentations; overall urine culture volume per year; proportion of all positive urine cultures representing ASB; and overall ASB antibiotic use, calculated as the ratio of ASB treated with antibiotics to all positive urine cultures.

**Results:**

The annual volume of urine cultures decreased by approximately 25% (Figure 1), from 87,556 in 2017 to 65,955 in 2023. The percentage of positive cultures representing ASB is depicted in Figure 2. The ASB smart phrase was documented in the medical record of 233 patients over one year: 165 (71%) were women, with a median age of 72 [48, 82] years. Emergency medicine providers utilized the smart phrase most frequently (97/233, 42%), followed by consultants (79/233, 34%). Overall ASB antibiotic use ranged from 9.7-22.7% in Q1 2022 and declined to 4.5-7% in Q1 2024. The primary endpoint, the system-wide average treatment of patients with ASB, declined modestly over time, but improvements were inconsistent among hospitals (Figure 3).

**Conclusion:**

Ongoing interventions targeting diagnostic and antibiotic stewardship through educational efforts were associated with a reduction in testing and treatment of ASB. However, suboptimal treatment of ASB remain a concern.

**Disclosures:**

**Rachel M. Kenney, PharmD, BCIDP**, Medtronic Inc: Spouse is an employee, stockholder **Erin Eriksson, PharmD, BCPS, BCIDP**, Stryker Corp: Stocks/Bonds (Public Company)

